# Male circumcision and HIV infection among sexually active men in Malawi

**DOI:** 10.1186/s12889-015-2384-z

**Published:** 2015-10-13

**Authors:** Namuunda Mutombo, Beatrice Maina, Monica Jamali

**Affiliations:** African Population and Health Research Center (APHRC), P.O Box 10787, 00100 Nairobi, Kenya; APHRC, Nairobi, Kenya; Chancellor College, University of Malawi, Zomba, Malawi

**Keywords:** HIV infection, HIV prevention, Malawi, Male circumcision, HIV risk

## Abstract

**Background:**

The HIV epidemic remains a major health challenge all over the world. In 2013, an estimated 35million people were living with HIV globally. Male circumcision is increasingly being adopted as a method of HIV prevention. WHO and UNAIDS have advised that male circumcision be added to current HIV interventions. Malawi is one of the countries hardest hit by HIV/AIDS with a prevalence rate of 11 % and male circumcision prevalence of 21.6 % in 2010. Prior to 2011, traditional male circumcision in Malawi was the dominant form of male circumcision, mainly for cultural and religious reasons. This paper looks at male circumcision as a prevention method against HIV by examining the relationship between male circumcision and HIV status among Malawian men.

**Methods:**

The data used were collected as part of the 2010 Malawi Demographic and Health Survey. The methodology used in the 2010 MDHS has been comprehensively described by the National Statistical Office of Malawi and ICF Macro. Our analysis is based on men aged 15–54 years who were tested for HIV and responded to questions on circumcision during the survey. Sixty one percent of the 7175 men interviewed in the MDHS, qualified for this analysis. The sample was weighted to ensure representativeness. Frequencies, cross-tabulations, univariate and multivariate logistic regressions were conducted. Differences in the prevalence of HIV infection among circumcised and uncircumcised men were determined with Chi-squared tests.

**Results:**

There is no significant difference in HIV prevalence between circumcised (12 %) and uncircumcised men (10 %). Among circumcised men, age and number of lifetime partners are the dominant correlates of HIV status. Additionally, circumcised men who have had ritual sex are two times more likely (OR = 2.399) to be HIV+ compared to circumcised men who have never had ritual sex.

**Conclusion:**

This study has demonstrated that traditional male circumcision was not associated with HIV infection in pre-2010 Malawi. Among circumcised men, age and number of lifetime partners are correlates to HIV status while circumcised men who have had ritual sex are more likely to be diagnosed with HIV than circumcised men who have not had ritual sex.

## Background

The HIV epidemic remains a major health challenge all over the world. In 2013, the UNAIDS estimated 35million people were living with HIV globally; and in the same year, 2.1 million new HIV infections and 1.5 million AIDS related deaths occurred [1]. Recent studies have identified approaches for HIV prevention especially those that target the generalized epidemic in sub-Saharan Africa and includes Comprehensive condom and lubricant programming, harm reduction for people who inject drugs, behavioral interventions, prevention of transmission in health-care settings, ARV-related prevention, and voluntary medical male circumcision for HIV prevention [2–7]. Results from randomized clinical trials conducted in Kenya, Uganda and South Africa established that male medical circumcision greatly reduced the risk of HIV infection among men [3, 8, 9]. Further studies have shown how those already infected can be treated [2, 10] or how the effects of the virus on a person’s immunity can be minimized by avoiding reinfection [8, 11]. As a result of the findings of the randomized clinical trials which showed that medical male circumcision can be used as a prevention measure for HIV infection [3, 8, 9], the World Health Organization (WHO) and the Joint United Nations Programme on HIV/AIDS (UNAIDS) recently recommended that circumcision be added to current interventions to reduce the spread of HIV [12, 13]. Male circumcision is defined as the complete removal of the foreskin [14]. Traditionally, many African societies have embraced male circumcision as a rite of passage from childhood to adulthood, rather than a medical operation [14] and also for religious and to a lesser extent, for medical purposes [15]. While not all communities practice circumcision, studies investigating HIV transmission done among communities practicing circumcision have consistently found that circumcision significantly reduces the rate of HIV transmission at the community level [16, 17].

In many of these studies, the outcome has been that circumcised men are significantly less likely to acquire HIV when exposed sexually to HIV-positive women as compared to men with intact foreskins [3, 8, 9, 18]. Special phagocytic cells in the foreskin, Langerhans cells, are said to act as magnets to the virus which attaches to the cell and thus enters the body [19]. A recent review by Morris and Wamai termed the inner foreskin as a mucosal epithelium which is deficient in protective keratin but rich in HIV target cells [20]. Once exposed to infected mucosal secretions, infected T-cells combine with keratinocytes and then transfer HIV to Langerhans cells via dendrites just under the surface of the inner foreskin which then migrates to the basal epidermis and then pass HIV to T-cells leading to infection [20]. This is aggravated by occurrence of any inflammations and ulceration and also tearing of the foreskin during intercourse [20]. In addition, randomized trials of male circumcision showed that male circumcision significantly reduced the bacterial load by reducing both the prevalence and abundance of many coronal sulcus bacteria [21]. Nevertheless, male circumcision does not provide a complete prevention against HIV infection, and circumcised men can become infected, and can transmit the virus to their partners [2, 3, 8, 9].

However, there still remain gaps and unanswered questions relating to HIV transmission and circumcision. Although not supported by literature, one major concern is the behavioral disinhibition where circumcised men could feel more protected against HIV infection and hence may engage in a riskier behavior. Traditionally, and in some communities, young men are taken into seclusion and given advice touching on their responsibilities which includes their sexual behavior [14]. Though not well documented, its often assumed that these ‘guidance and counseling’ sessions inform or instill behavior change and risk reduction on HIV transmission among circumcised men as compared to the uncircumcised, who principally do not go through such sessions. However, cluster randomized trials done in South Africa to assess the interventions that could prevent men from sexual risky behaviors after circumcision showed that social cultural values dictated how men should behave among traditionally circumcised men [22] and hence resistance to change; while among those medically circumcised, a focused counseling session had short term effects on reducing risky sexual behavior [23]. During male circumcision rituals, the period of seclusion constitutes the most significant part where new attitudes, practices and behaviours are learnt which includes sexual issues [14]. Whereas sexual reserve and inappropriateness of promiscuity after reintegration into society are emphasized, in other contexts, boys are encouraged to have sex to prove their manhood even during the seclusion period, often referred to as ritual sex [14]. Ritual sex is practiced in many African communities and is associated with traditional circumcision where men are “cleansed through” and/or “trained on” sexual activities. Although ritual sex is conducted in some communities in Malawi, there is limited information regarding this practice. However, in Zambia, ritual sex is associated with traditional circumcision and use of condom is not emphasized [24, 25]. There is therefore a need to look at the association between type of circumcision and HIV infection in Malawi, as well as analyze the effect of ritual sex on HIV infection.

While some studies show that pre-pubertal circumcision is associated with reduced risk of HIV infection [26, 27], one study shows no significant association between age at circumcision and HIV infection [28]. Male circumcision in many African societies is done at puberty [29] ; however, there are communities that do not have a defined circumcision period and therefore some men are circumcised as infants, while others are circumcised at adulthood. When circumcised at old age, these men are already exposed to sexual activities for a long time, and have higher chances of being exposed to HIV as compared to younger men.

### Study setting and objectives

Malawi is one of the countries hardest hit by HIV/AIDS. Though recent indicators have shown some decline from a prevalence rate of 11.8 to 10.6 % in 2004 and 2010 respectively, a lot still needs to be done [30]. The 2012 AIDS Response Progress Report shows that Malawi has registered progress in the areas of prevention; treatment, care and support; and impact mitigation [30]. However, the country has been criticized for being slow in the introduction of free male circumcision in public facilities and only recently, in 2011, the Malawi government adopted a policy on medical male circumcision as part of its HIV prevention strategy, offering the service free at public hospitals [30]. But male circumcision has been in practice in Malawi for cultural and religious reasons [31] and only recently that men are embracing circumcision and mainly due to its medical advantage. According to WHO, voluntary medical male circumcision (VMMC) in Malawi has been rising slowly from less than 600 men in 2008 to over 40,000 in 2013 and have obtained almost 4 % progress towards achieving the 80 % coverage by 2016 [32, 33]. Even though male circumcision is viewed as one of the key interventions on HIV prevention, no study has examined its effect within the Malawian context. The objective of this study is to evaluate the relationships between male circumcision and HIV status among Malawian men before the implementation of VMMC using data from the 2010 Malawi Demographic and Health Survey (MDHS).

## Methods

### Data source and sample size

This paper uses cross-sectional data from the nationally representative sample of the 2010 MDHS Men’s File to examine the association between male circumcision and HIV status in Malawi. The methodology used in the 2010 MDHS has been comprehensively described by the National Statistical Office of Malawi and ICF Macro [34]. The primary objectives of the 2010 MDHS project were to provide up-to-date information on fertility levels; nuptiality; sexual activity; fertility preferences; awareness and use of family planning methods; breastfeeding practices; nutritional status of mothers and young children; early childhood mortality; maternal mortality; maternal and child health; malaria; awareness and behavior regarding HIV/AIDS and other sexually transmitted infections; and HIV prevalence. This was a nationally representative survey whose sample design was tailored to provide specific indicators at national and regional levels. A total of 23,020 women (15–49) and 7175 men (15–54) were interviewed from 24,825 households using the Woman’s and Man’s Questionnaires, respectively. This paper is based on men (15–54) who tested for HIV and responded to questions on circumcision during the survey. Out of the 7175 men interviewed in the MDHS, 4358 (61 %) men were valid for this analysis. The sample was weighted using the DHS generated survey weights to ensure representativeness.

### Ethics statement

The 2010 MDHS was approved by Malawi Health Sciences Research Committee, the Institutional Review Board of ICF Macro, and the Centre for Disease Control and Prevention (CDC) in Atlanta. Use of the MDHS data was communicated to and permission to use granted by Measure DHS.

### Variables

The dependent variable used is HIV status. This variable is measured as a dichotomous variable and is coded as negative status and positive status. Four models were applied to assess the correlates of HIV status. These models are: I) unadjusted analysis of HIV correlates among circumcised and uncircumcised men; II) adjusted analysis of HIV correlates among circumcised and uncircumcised men; III) adjusted analysis of HIV correlates among circumcised men; and IV) adjusted analysis of HIV correlates among uncircumcised men.

The main independent variable is the circumcision status i.e. circumcised or not circumcised. The control variables used in the study are: age, marital status, age at sexual debut, ever had ritual sex, number of lifetime partners, type of residence, region of residence, religion, level of education and wealth index. The confounding factors in the study are age at circumcision, and type of circumcision, which are only used in Model IV as they only apply to the circumcised group. The wealth index was constructed from the wealth quintile as follows: 1st and 2nd quintiles (poor); 3rd quintile (medium); and 4th and 5th quintiles (rich).

### Data analysis

In order to ascertain the effect of circumcision on HIV status among Malawian men (15–54), we used the Statistical Package for Social Sciences (SPSS) to generate frequencies, cross-tabulations and binary logistic regressions. The Chi-Square test was used to determine the level of association between the dependent and independent variables.

The odds ratio is used to depict the likelihood of a respondent testing positive for HIV. A ratio greater than 1 means greater likelihood of being HIV-positive than the reference category; a ratio less than 1 means lower likelihood of being HIV-positive than the reference category; and a ratio of 1 means same likelihood of being HIV-positive as the reference category. In this study, an independent variable is considered significant if its effect on the dependent variable (HIV status) is statistically significant at the 0.95 confidence interval (i.e. *p* ≤ 0.050).

## Results

### Characteristics of respondents

Table 1 below shows distribution by selected background characteristics of men who responded to the circumcision question and had their blood sample taken for HIV testing. Almost 80 % of the men were aged below 40 years; about 41 % were never married; almost 97 % initiated sex when aged below 25 years and one in every 25 men had participated in a ritual sex. Table 1 also shows that 83 % of the men had more than one life time sex partners.

**Table 1:** Distribution of respondents by selected background characteristics

Background characteristics	Sample size	
	%	*n*
*Age*		
15–24	35.2	1,533
25–39	43.9	1,911
40+	20.9	914
*Marital status*		
Never married	40.8	1,778
Currently married	55.3	2,410
Formerly married	3.9	170
*Age at sexual debut*		
< 15	21.0	915
15–24	75.6	3,294
25 + ^†^	3.4	149
*Ever had ritual sex*		
Yes	3.6	159
No	96.4	4,199
*Number of life time partners*		
1	17.2	750
2	24.8	1,082
3	21.3	921
4	11.2	486
5	8.5	371
6–9	9.0	394
10+	8.1	354
*Type of residence*		
Urban	14.8	647
Rural	85.2	3,711
*Region*		
Northern	17.2	750
Central	36.5	1,592
Southern	46.3	2,016
*Religion*		
Catholic	22.6	984
Other Christian	67.2	2,930
Muslim	10.2	444
*Level of education*		
None	5.8	253
Primary	61.2	2,669
Secondary+	33.0	1,436
*Wealth index*		
Poor	34.3	1,496
Medium	20.7	903
Rich	45.0	1,959
*Circumcision status*		
Circumcised	19.1	831
*Age at circumcision*		
< *10*	*26.4*	*219*
*10-14*	*52.0*	*432*
*15+*	*21.6*	*180*
*Type of circumciser*		
*Traditional*	*85.4*	*710*
*Medical*	*14.6*	*121*
Not circumcised	80.9	3,527
*HIV status*		
Negative	89.9	3,918
Positive	10.1	440
Total	**100.0**	**4,358**

Slightly over 85 % of men in the sample lived in rural areas with the Southern region having the highest percentage of men at 46 %, while the Northern region had the least at 17 %. Almost 90 % of Malawian men were Christians, 61 % had primary level education and only 34 % were regarded as poor. On circumcision, over 80 % of Malawian men reported to have been uncircumcised at the time of the survey. Among the circumcised men, data show that most circumcised men (85 %) underwent traditional circumcision while the rest (15 %) underwent medical circumcision. The data further show that the majority (52 %) were circumcised between the ages of 10 and 14 while a quarter were circumcised before they were 10 years old. The rest (22 %) got circumcised at 15+. Results of HIV testing showed that 10 % of Malawian men were HIV positive at the time of the survey.

### Bivariate results

Results in Table 2 show men’s HIV status by background characteristics. Overall, 10 % of men were HIV positive. Older men (40+), formerly married, men with more than four lifetime partners were more likely to be HIV positive compared to their counterparts (*P* < 0.001). Urban areas, and the southern region had significantly higher proportion of HIV + men (*P* < 0.001) compared to rural areas and Northern and Central regions respectively. The rich also were more likely to be HIV+ as compared to medium rich and the poor (*P* < 0.001). Similarly, men who initiated sex when they were aged more than 24 years also were more likely to be HIV + (*P* < 0.01). A significantly higher proportion of HIV+ men was also observed among the circumcised (*P* < 0.05) compared to the uncircumcised.

**Table 2:** Percent distribution of respondents by HIV status and selected background characteristics

Background characteristics	HIV status		Sample size
	Negative	Positive	
Age***			
15–24	97.8	2.2	1533
25–39	87.5	12.5	1911
40+	81.1	18.9	914
Marital status***			
Never married	94.0	6.0	1778
Currently married	87.8	12.2	2410
Formerly married	76.3	23.7	170
Age at sexual debut**			
< 15	92.7	7.3	915
15-24	89.2	10.8	3294
25 + ^†^	87.7	12.3	149
*Ever had ritual sex*			
Yes	86.4	13.6	159
No	90.1	9.9	4199
Number of life time partners***			
1	98.0	2.0	750
2	92.7	7.3	1082
3	89.3	10.7	921
4	91.6	8.4	486
5	85.2	14.8	371
6-9	80.7	19.3	394
10+	79.7	20.3	354
Type of residence***			
Urban	84.8	15.2	647
Rural	91.4	8.6	3711
Region***			
Northern	93.2	6.8	750
Central	91.9	8.1	1592
Southern	87.2	12.8	2016
*Religion*			
Catholic	89.9	8.4	984
Other Christian	89.8	8.6	2930
Muslim	90.4	7.7	444
*Level of education*			
None	87.5	12.5	253
Primary	90.3	9.7	2669
Secondary+	89.7	10.3	1436
Wealth index***			
Poor	92.2	7.8	1496
Medium	91.7	8.3	903
Rich	87.7	12.3	1959
Circumcision status*			
Circumcised	87.9	12.1	831
Not circumcised	90.5	9.5	3,527
Total	**89.9**	**10.1**	**4,358**

*p < 0.050; **p < 0.01; ***p < 0.001

### Multivariate results

Four regression models were fitted (Table 3). Model I is the unadjusted model while model II, III and IV are adjusted models representing both uncircumcised and circumcised men, uncircumcised men only and circumcised men only, respectively. Model III and IV were used to depict the most influential variables among uncircumcised and circumcised groups. The unadjusted model shows that 8 out of the thirteen variables are significant on the likelihood of being HIV+. These variables are: age, marital status, age at sexual debut, number of lifetime sexual partners, residence, region, wealth index and circumcision status. However, when we control for other factors (Model II), only four variables remain significant on the likelihood of being HIV+. These are age, number of lifetime sexual partners, residence and region.

**Table 3:** Respondent’s odds of being HIV-positive by selected background characteristics

Background characteristics	Model I		Model II		Model III		Model IV	
	Exp β	CI	Exp β	CI	Exp β	CI	Exp β	CI
*Age*								
15–24	0.097***	.072–.132	0.131***	.085-.200	0.149***	.092-.240	0.070***	.025-.197
25–39	0.634***	.525-.766	0.675**	.538-.848	0.610***	.610-.796	0.895	.557-1.437
40+^a^	-	-	-	-	-	-	-	-
*Marital status*								
Never married	0.356***	.293-.432	1.016	.778-1.326	1.010	.742-1.375	1.149	.640-2.065
Ever Married^a^	-	-	-	-	-	-	-	-
*Age at sexual debut*								
< 15	0.410***	.327-.513	0.875	.656-1.167	0.889	.637-1.242	0.745	.407-1.366
15+^a^	-	-	-	-	-	-	-	-
*Ever had ritual sex*								
Yes	0.706	.463-1.075	1.172	.724-1.898	0.822	.434-1.556	2.399*	1.053-5.466
No^a^	-	-	-	-	-	-	-	-
*Number of life time partners*								
1	0.103***	.068-0.156	0.173***	.099-.301	0.117***	.059-.234	0.500	.184-1.356
2	0.351***	.274-.449	0.423***	.319-.562	0.438***	.317-.605	0.314***	.165-.599
3	0.586***	.468-.734	0.605***	.466-.786	0.582***	.430-.787	0.513*	.286-.921
4	0.447***	.327-.610	0.454***	.318-.649	0.378***	.243-.589	0.705	.365-1.363
5^a^	-	-	-	-	-	-	-	-
*Type of residence*								
Urban	1.912***	1.588-2.303	2.024***	1.567-2.615	2.311***	1.715-3.112	1.652	.916-2.908
Rural^a^	-	-	-	-	-	-	-	-
*Region*								
Central/Northern	0.537***	.452-.639	0.591***	.476-.733	0.573***	.450-.729	0.719	.415-1.246
Southern^a^	-	-	-	-	-	-	-	-
*Religion*								
Catholic	1.107	.807-1.518	1.443	.931-2.237	0.820	.269-2.503	2.426*	1.228-4.793
Other Christian	1.131	.855-1.498	1.441	.971-2.137	0.873	.291-2.623	1.300	.805-2.098
Muslim^a^	-	-	-	-	-	-	-	-
*Level of education*								
None	1.111	.786-1.570	1.262	.799-1.993	2.438**	1.434-4.144	0.395	.150-1.035
Primary	0.889	.738-1.072	1.194	.932-1.528	1.342*	1.007-1.787	0.952	.540-1.679
Secondary + ^a^	-		-	-	-	-	-	-
*Wealth index*								
Poor	0.575***	.468-.707	0.787	.598-1.036	0.753	.546-1.040	1.123	.629-2.004
Medium	0.788*	.630-.988	0.757	.562-1.019	0.606**	.422-.871	1.928*	1.057-3.517
Rich^a^	-	-	-	-	-	-	-	-
*Circumcision status*								
Circumcised	1.341**	1.103-1.631	1.177	.871-1.590	-	-	-	-
Not circumcised^a^	-	-	-	-	-	-	-	-
*Age at circumcision*								
< 10	0.879	.531-1.455	-	-	-	-	1.433	.737-2.787
10-14	0.855	.549-1.332	-	-	-	-	1.409	.787-2.520
15+^a^	-	-	-	-	-	-	-	-
*Type of circumciser*								
Traditional	1.025	.641-1.637	-	-	-	-	0.901	.478-1.699
Medical^a^	-	-	-	-	-	-	-	-
Constant	-	-	0.271***	-	0.457	-	0.209**	-

*p < 0.050; **p < 0.01; ***p < 0.001; ^a^Reference category

Men aged below 40 years were found to be strongly associated with decreased odds of being HIV+ compared to men aged 40 years and above. Men who had up to 4 lifetime sexual partners had lower odds of being HIV+ compared to those who had 5 or more lifetime sexual partners, while those residing in the urban areas were at least two times more likely to be HIV+ than their rural counterparts. Compared to men from the Southern region, men from the Northern and Central regions were almost 50 % likely to be HIV+. Even though circumcision was significant in Model I, it was not significant after controlling for other variables.

Table 3 also shows that the four significant variables in Model II produce similar effects in Model III. However, two additional variables, namely education and wealth index, are also significant on the probability of being diagnosed with HIV among the uncircumcised men: the higher the level of education the lower the odds of being HIV+ while the level of wealth is directly related with the odds of being HIV+. In the fourth model, two more variables (ritual sex and religion) that were not significant in the other three models show strong association with the likelihood of being diagnosed with HIV among the circumcised men. Circumcised men who had ritual sex were at least 2 times more likely to be HIV+ compared to those who had not participated in ritual sex. A further analysis of ritual sex was done (data not shown in tables) to show the rates of ritual sex among the traditionally versus medically circumcised. The results showed that among circumcised men, reported ritual sex was 3.9 %. Among those circumcised traditionally, 4.4 % reported having had ritual sex compared with 1.4 % reported among the medically circumcised. Circumcised men of Catholic faith had at least 2 times higher odds of being HIV+ compared to Muslim men who have undergone circumcision.

The other significant factors are age, number of lifetime sexual partners and the wealth index. While age and lifetime sexual partners influence circumcised men in the same way as they influence other models, the wealth index influences circumcised men differently: circumcised men who are in the middle tertile had higher odds of being HIV+ compared to the rich men.

## Discussion

Though statistically insignificant, our paper shows that circumcised men in Malawi have a higher HIV prevalence rate (12 %) than the uncircumcised men (10 %). However, the circumcision in this context is mainly traditional circumcision as voluntary medical male circumcision was introduced in 2011, a year after these data were collected. This is contrary to the literature, which suggests that even traditional male circumcision is highly effective in HIV prevention [35]. However, our general model shows that other factors exhibited significant influence on the HIV status. These are age, marital status, number of lifetime sexual partners, type of residence and region. The mode of HIV transmission in Malawi is predominantly heterosexual and as such, it is also expected that individuals with higher levels of exposure to sexual intercourse would be more prone to HIV infection than those with lower levels of exposure to sexual intercourse. While our findings on age and number of lifetime sexual partners appear to confirm this assertion, our result on the effect of marital status deviates from this assertion. The never married are 1.3 times more likely to be HIV+ than the ever married men. Even though our study did not measure the actual number of times an individual had sexual intercourse, it is often assumed that the married have a higher coital frequency than the unmarried persons. It is also not very clear why men in the Northern and Central Regions have higher HIV prevalence than their counterparts in the Southern Region. However, urban areas have in many cases been found to have a higher HIV prevalence rate than rural areas [36, 37] and this is the case for Malawi as well.

Among the circumcised men (Model IV), age and number of lifetime sexual partners remain dominant as correlates of HIV status. Interestingly, however, this model appears to provide clues as to why there is no significant difference between circumcised and uncircumcised men in terms of HIV status. Circumcised men who ever had ritual sex are 2.4 times more likely to be diagnosed with HIV than circumcised men who have never had ritual sex. The context of ritual sex in Malawi is not immediately clear. However, evidence of ritual sex after traditional circumcision has been documented in Zambia, one of Malawi’s neighboring countries [24, 25]. This ritual sex is associated with traditional circumcision and exposes men to the risk of HIV infection as use of condom is not emphasized thereby negating the preventive role that male circumcision is supposed to play [24]. Since most of the circumcised men were circumcised by traditional methods in Malawi, there is a high likelihood that the observations made in Zambia [25] could also be at play.

Model IV also confirms observations from elsewhere that Catholics/Christians have higher HIV prevalence than Muslims [38]. Interestingly, circumcised men in the medium wealth index (or the middle class) have significantly higher odds of being diagnosed with HIV than the rich circumcised men. This seems paradoxical as rich men are often viewed as agents of HIV [39] as they are often associated with concurrent multiple sexual relationships [40].

There are some limitations associated with DHS data and that in a way may impact the results of this study. First, DHS data are cross-sectional and hence temporality of the association between male circumcision and HIV infection cannot be absolutely established. Secondly, male circumcision is self-reported, and thirdly, it’s impossible to establish when men were circumcised and when they became HIV+ and lastly is the dearth of information around ritual sex and traditional circumcision in Malawi.

## Conclusions

Our study shows that there is a weak association between male circumcision and HIV infection in Malawi. Twelve percent of circumcised men were HIV positive compared to almost 10 % of uncircumcised men. However, most of the circumcised men in this study were circumcised traditionally as the policy to offer medical male circumcision was only introduced in 2011 (a year after the 2010 MDHS was conducted); and hence the results of this study refer mainly to traditional circumcision and should be interpreted with a lot of caution. In addition, the finding could be a pointer for the increased need for voluntary medical male circumcision in Malawi, and the need to shun away from the (unsafe) traditional male circumcision practices. Given that most circumcised males were circumcised using traditional methods and that HIV-positive status among circumcised men is significantly associated with ritual sex, it can be deciphered that there is a strong association between traditional circumcision (which may include ritual sex) and HIV infection in Malawi which may be connected to the actual reasons why HIV prevalence is higher among circumcised men than uncircumcised men in Malawi. Therefore, our study shows that traditional male circumcision does not act as a preventive measure to HIV infection in Malawi. However, we acknowledge that the main limitation of this notion is the dearth of information around ritual sex and traditional circumcision in Malawi.
